# In vitro antagonistic inhibitory effects of palm seed crude oils and their main constituent, lauric acid, with oxacillin in *Staphylococcus aureus*

**DOI:** 10.1038/s41598-020-80481-0

**Published:** 2021-01-08

**Authors:** Klara Lalouckova, Eva Skrivanova, Johana Rondevaldova, Adela Frankova, Josef Soukup, Ladislav Kokoska

**Affiliations:** 1 Department of Microbiology, Nutrition and Dietetics, Faculty of Agrobiology, Food and Natural Resources, Czech University of Life Sciences Prague, 165 00 Praha 6-Suchdol, Czech Republic; 2 Department of Nutritional Physiology and Animal Product Quality, Institute of Animal Science, 104 00 Praha-Uhrineves, Czech Republic; 3 Department of Crop Science and Agroforestry, Faculty of Tropical AgriSciences, Czech University of Life Sciences Prague, 165 00 Praha 6-Suchdol, Czech Republic; 4 Department of Food Science, Faculty of Agrobiology, Food and Natural Resources, Czech University of Life Sciences Prague, 165 00 Praha 6-Suchdol, Czech Republic

**Keywords:** Microbiology, Diseases, Infectious diseases

## Abstract

Infections caused by *Staphylococcus aureus* are a serious global threat, and with the emergence of antibiotic resistance, even more difficult to treat. One of the possible complications in antistaphylococcal therapy represents negative interactions of antibiotics with food. In this study, the in vitro interaction between oxacillin and crude palm seed oil from *Astrocaryum vulgare*, *Cocos nucifera*, and *Elaeis guineensis* against nine strains of *S. aureus* was determined using the checkerboard method. Lauric acid was identified as a major constituent of all tested oils by gas chromatography. The results showed strong concentration dependent antagonistic interactions between palm oils and oxacillin with values of fractional inhibitory concentrations indices ranging from 4.02 to 8.56 at concentrations equal or higher than 1024 µg/mL of the tested oils. Similarly, lauric acid in combination with oxacillin produced antagonistic action with fractional inhibitory concentration indices ranging from 4.01 to 4.28 at 1024 µg/mL. These findings suggest that interference between oxacillin and palm oils and their constituents can negatively affect the treatment of staphylococcal infections in humans and other animals.

## Introduction

*Staphylococcus aureus*, a gram-positive bacterium, occurs naturally on the skin and mucus membranes of healthy individuals and is a common cause of pneumonia, skin infections, and systemic infections in humans and other animals^1^. Its ability to resist a broad range of antibacterials in a short period makes it one of the most dangerous microorganisms influencing the global population, with strains resistant to beta-lactams, such as methicillin-resistant *S. aureus* (MRSA) being considered a high priority pathogen^2^. However, currently used antistaphylococcal antibiotics (e.g., vancomycin, daptomycin, and linezolid) are struggling against rising resistance, serious side effects, and relatively high costs^3^. Hence, steps toward elimination of bacteremia caused by multi-drug resistant strains are still needed. Among other possible options, combinatory treatment is valuable for eradicating evolution of resistance by targeting different sites of the bacterial cell^4,5^. For example, the combination of vancomycin and a beta-lactam antibiotic (oxacillin) is currently being used effectively in the treatment of MRSA^6^. A combination of beta-lactam antibiotic and beta-lactamase inhibitors, such as co-amoxiclav consisting of amoxicillin and clavulanic acid, has also been used for MRSA therapy^7^. However, combinations of certain antibiotics can produce negative interactions and undesirable side effects. Antagonistic action generally occurs in the treatment of infection when mixing bactericidal and bacteriostatic drugs^8^, as seen when using a combination of the slow acting bactericidal vancomycin and bacteriostatic clindamycin against *S. aureus*^9^. Generally faster acting agents, such as clindamycin or oxacillin, inhibit the function of vancomycin, which has a gradual onset of action and exhibits antibacterial properties only on replicating cells^10^.

Combining various drugs is the prevalent praxis used to obtain an increase of the desired effects, such as in anesthesia and pain management. However, enhancement of the undesired effects might also occur, limiting the therapeutic value^11^. Such interactions are also known to occur when combining drugs with various foods and food products. Drug-food interactions are defined as changes in efficacy and/or toxicity of pharmaceutical drugs induced by the consumption of any food product, including functional foods and dietary supplements^12^. Various drug–food interactions (e.g., drug interaction with the fat content of the meal), drug–nutrient interactions (e.g., with grapefruit juice or soy) and herb–drug interactions (e.g., with ginkgo or St John’s wort) have been described and reviewed^13–16^. The most well-known example of drug–food interaction is that of grapefruit juice that can inhibit the intestinal metabolism of more than 85 drugs by altering cytochrome P450 (CYP3A4) isoforms^17^. Grapefruit juice, combined with erythromycin, increases the bioavailability of the antibiotic in the small intestine^18^, thereby increasing the possibility of adverse effects, such as cardiac dysrhythmias^19^. In contrast, a combination of ampicillin and pomegranate methanol extract acts synergistically in vitro against MRSA^20^. Similarly, fatty acid methyl esters obtained from soybean, corn, and sunflower crude oils potentiate the antifungal effect of itraconazole in vitro^21^.

Vegetable oils are essential components of animal nutrition and contain various biologically active constituents such as carotenoids, tocopherols^22^, coenzyme Q10^23^, and fatty acids^24^. These are carboxylic acids with long, unbranched carbon chains, some of which may contain double bonds^25^. In general, fatty acids are believed to be responsible for the antibacterial activity of palm oils. They usually occur in the form of triglycerides; their hydrolysis into free fatty acids is necessary for their antibacterial effect to be exerted^26–28^. The seeds of tropical palms such as tucuma (*Astrocaryum vulgare*), coconut (*Cocos nucifera*), and African oil (*Elaeis guineensis*) palm are one of the most economically important sources of plant oils, and are known to contain mainly fatty acids of medium-chain length (MCFAs), with a prevalence of lauric acid (C_12:0_; LA). Apart from various physiological functions, the LA produces a growth-inhibitory effect against algae^29^, fungi^30^, protozoa^31^, and both gram-negative^32–34^ and gram-positive bacteria^35^. It also inhibits the production of bacterial virulence factors such as beta-lactamases, a group of exoproteins inducing beta-lactams inactivation and moreover enhancing toxin production by *S. aureus* at subinhibitory antibiotic concentrations^36^; toxic shock syndrome toxins^37^; and hemolysins^38^. In addition to LA, tropical palms contain caproic (C_6:0_), caprylic (C_8:0_), and capric (C_10:0_) acids that also possess various antimicrobial properties^39–41^. In addition, MCFAs are known to be effective against *S. aureus*. Batovska et al.^42^ concluded that when these fatty acids are esterified with glycerol and create e.g., monolaurin, their direct antistaphylococcal effect is intensified. Ubgogu et al.^43^ observed a noticeable in vitro inhibitory effect of *E. guineensis* palm kernel oil against *S. aureus*. The mechanism of antimicrobial action of fatty acids is not fully known, the prime target seems to be the bacterial cell membrane together with various essential processes that occur within and at the membrane, including nutrient uptake or enzyme inhibition^44^. The amphiphilic nature of fatty acids enables them to act as detergents at high concentrations and aid the solubilization of the lipids in the membranes^45^. It has been experimentally proven that doses of LA equal to or higher than 100 µg/mL (≥ 500 µM) induce reversible morphological changes of lipid bilayers^46^, cause partial solubilization of the cell membrane, and interfere with metabolic regulation, leading to the inhibition of bacterial growth^44^. It is well known that bactericidal drugs are most potent with actively growing cells and that inhibition of growth, induced by a combination with a bacteriostatic drug, can result in reduction of drug efficacy^8^.

Besides the antimicrobial properties, LA is known to exhibit synergistic antistaphylococcal activity in combination with monolaurin^42^, lactic acid^26^, and gentamicin^47^. However, limited information is available regarding the possible negative interactions of palm oils with antimicrobial agents. Therefore, we decided to perform a screening test, focused on determining the combined effect of the chosen palm oils and free MCFAs (C_6:0_–C_12:0_) with representatives of all major antibiotic groups, namely beta-lactams (amoxicillin, ampicillin, and oxacillin), tetracyclines (tetracycline), glycopeptides (vancomycin), and aminoglycosides (gentamicin), against three reference strains of *S. aureus*. Among the free MCFAs, only LA showed above mentioned synergism with gentamicin against chosen *S. aureus* strains; the interactions of all free MCFAs with tetracycline and vancomycin were indifferent, but beta-lactams, namely amoxicillin, ampicillin and especially oxacillin, showed results that were warranted further investigation with LA showing the strongest antagonistic interactions (Lalouckova and Kokoska, unpublished data). In the present study, we evaluated in vitro combinatory effect of *A. vulgare*, *C. nucifera*, and *E. guineensis* seed crude oils and their main constituent LA with oxacillin, using the checkerboard microdilution method, against different strains of *S. aureus*.

## Results

### Fatty acid composition of crude oils

In the first part of the study, fatty acid composition of the tested oils was identified using GC-FID. As shown in Table 1, oils from *A. vulgare*, *C. nucifera*, and *E. guineensis* consisted mainly of 58.2, 53.88 and 52.24 mg/g of MCFAs, respectively. LA was a major constituent of the oils, present at a concentrations of 53.37 mg/g in *A. vulgare*, 45.24 mg/g in *E. guineensis*, and 41.31 mg/g in *C. nucifera* oil. This profile corresponded with the total saturated fatty acid composition, where *A. vulgare* is followed by *C. nucifera* and *E. guineensis* oil with values of 90.32, 82.35, and 80.21 mg/g, respectively. The contents of other MCFAs, namely caprylic, capric, and caproic acids, were 6.73, 5.29, and 0.55 mg/g in *C. nucifera*, 3.48, 3.27, and 0.25 mg/g in *E. guineensis*, and 2.47, 2.15, and 0.21 mg/g in *A. vulgare* oil, respectively. In addition to MCFAs, saturated fatty acids consisted of myristic, palmitic, and stearic acids. Their respective contents were 24.82, 5.41, and 1.89 mg/g in *A. vulgare*; 16.5, 9.05, and 2.92 mg/g in *C. nucifera*; and 15.85, 9.46, and 2.66 mg/g in *E. guineensis* oil.

**Table 1:** Fatty acid profile of crude palm seed oils.

	Fatty acids content (mg/g)/plant species		
	*Astrocaryum vulgare*	*Cocos nucifera*	*Elaeis guineensis*
**Saturated fatty acids (SFA)**			
Caproic (C_6:0_)	0.21	0.55	0.25
Caprylic (C_8:0_)	2.47	6.73	3.48
Capric (C_10:0_)	2.15	5.29	3.27
Lauric (C_12:0_)	53.37	41.31	45.24
Myristic (C_14:0_)	24.82	16.5	15.85
Palmitic (C_16:0_)	5.41	9.05	9.46
Stearic (C_18:0_)	1.89	2.92	2.66
Total SFA	90.32	82.35	80.21
Total MCFA	58.2	53.88	52.24
**Monounsaturated fatty acids (MUFA)**			
Palmitoleic (C_16:1_)	0.02	–	0.03
Oleic (C_18:1_)	6.47	11.72	16.54
Eicosenoic (C_20:1_)	0.05	0.15	0.10
Total MUFA	6.55	11.87	16.67
**Polyunsaturated fatty acids (PUFA)**			
Linoleic (C_18:2_)	2.77	4.79	2.68
α-Linoleic (C_18:3_)	0.06	0.88	0.03
Arachidonic (C_20:4_)	0.07	0.13	0.13
Total PUFA	2.9	5.8	2.84

Data are presented as average of two analyses, each performed in triplicate.

Although saturated fatty acids were the major constituents of all oils analysed, the composition of monounsaturated (MUFAs) and polyunsaturated (PUFAs) fatty acids was also determined. MUFAs, consisting of oleic, eicosenoic, and palmitoleic acids, were the most abundant in *E. guineensis* oil (16.67 mg/g), and were present at relatively lower concentrations in *C. nucifera* and *A. vulgare* oil (11.87 and 6.55 mg/g, respectively). The oleic acid content was the highest in all samples, ranging from 6.47 to 16.54 mg/g. In contrast, eicosenoic (0.05–0.15 mg/g) and palmitoleic acid (≤ 0.03 mg/g) were present in minor amounts.

PUFA content in *C. nucifera* oil (5.8 mg/g) was double that of *A. vulgare* (2.9 mg/g) and *E. guineensis* (2.84 mg/g) oil. In *C. nucifera* oil, linoleic acid was the most abundant PUFA, similar to *A. vulgare* and *E. guineensis* oil at 4.79, 2.77, and 2.68 mg/g, respectively. The second most abundant PUFA in *C. nucifera* oil was α-linoleic acid (1.88 mg/g), which was also present in lower amounts in *A. vulgare* (0.06 mg/g) and *E. guineensis* (0.03 mg/g) oil. Compared to *C. nucifera* and *A. vulgare* oil (both 0.13 mg/g), arachidonic acid was the least abundant PUFA in *E. guineensis* oil (0.07 mg/g).

### Antistaphylococcal antagonistic effect of crude oils and oxacillin

In the first step, the susceptibility of the three tested *S. aureus* strains to oxacillin and hydrolyzed seed oils of *A. vulgare*, *C. nucifera*, and *E. guineensis* was determined using broth microdilution method to evaluate the suitable starting concentrations for combined effect (MIC—minimum inhibitory concentration—values for all tested oils in non-hydrolyzed forms > 8192 µg/mL; data not shown). *A. vulgare* oil induced the strongest antistaphylococcal effect, with MIC values ranging from 240 to 356 µg/mL, followed by that of *C. nucifera* (MIC values from 241 to 512 µg/mL) and *E.* *guineensis* (MIC values from 427 to 512 µg/mL). The MIC values of oxacillin ranged from 0.72 to 56.89 µg/mL.

Further, analysis of the combined effect of tested seed crude oils and oxacillin was performed by the checkerboard method. The results showed some degree of antagonism in all tested strains at certain concentrations of combinations, with indices of fractional inhibitory concentration (FICI) ranging between 8.56 and 4.02. In addition, a consistently antagonistic, concentration-dependent effect of the selected palm oils at concentration of 2048 µg/mL, and in some cases 1024 µg/mL, was seen, when combined with oxacillin against all tested *S. aureus* strains. All other combinations showed an indifferent relationship between tested agents, as shown in Table 2.

**Table 2:** Combinatory effect of crude palm seed oils and oxacillin against *Staphylococcus aureus* determined by checkerboard method.

*S. aureus* strain	MIC of compounds alone (µg/mL)		OXA with *Astrocaryum vulgare* oil at concentration (µg/mL)															
			2048		1024		512		256		128		64		32		16	
	MIC OXA	MIC OIL	MIC OXA	FICI	MIC OXA	FICI	MIC OXA	FICI	MIC OXA	FICI	MIC OXA	FICI	MIC OXA	FICI	MIC OXA	FICI	MIC OXA	FICI
29213	1.67	242	0.06	**8.51**	0.06	**4.27**	0.06	2.16	0.23	1.20	0.44	0.80	1.11	0.93	2.22	1.47	2.89	1.80
43300	56.89	356	1	**5.78**	1	2.90	6	1.55	7	0.84	21.67	0.74	30.22	0.71	43.56	0.86	53.33	0.98
SA1	2	240	0.06	**8.56**	0.06	**4.30**	0.07	2.17	0.21	1.17	0.41	0.74	0.63	0.58	1.06	0.66	1.88	1

*S. aureus* strain	MIC of compounds alone (µg/mL)		OXA with *Cocos nucifera* oil at concentration (µg/mL)															
			2048		1024		512		256		128		64		32		16	
	MIC OXA	MIC OIL	MIC OXA	FICI	MIC OXA	FICI	MIC OXA	FICI	MIC OXA	FICI	MIC OXA	FICI	MIC OXA	FICI	MIC OXA	FICI	MIC OXA	FICI
29213	0.72	512	0.06	**4.09**	0.71	2.98	0.32	1.44	0.38	1.02	0.71	1.23	0.94	1.43	2.39	3.37	2	2.80
43300	49.78	327	1	**6.28**	1	3.15	1	1.59	6.89	0.92	14.22	0.68	21.33	0.62	28.44	0.67	32	0.69
SA1	2	241	0.06	**6.03**	0.06	3.03	0.08	1.54	0.28	0.89	0.58	0.67	1	0.69	1.33	0.76	2	1.05

*S. aureus* strain	MIC of compounds alone (µg/mL)		OXA with *Elaeis guineensis* oil at concentration (µg/mL)															
			2048		1024		512		256		128		64		32		16	
	MIC OXA	MIC OIL	MIC OXA	FICI	MIC OXA	FICI	MIC OXA	FICI	MIC OXA	FICI	MIC OXA	FICI	MIC OXA	FICI	MIC OXA	FICI	MIC OXA	FICI
29213	0.75	427	0.06	**4.88**	0.06	2.48	0.09	1.32	0.31	1.02	0.53	1	0.69	1.08	0.78	1.11	0.56	0.78
43300	56.89	512	1	**4.02**	1	2.02	7	1.12	15.78	0.78	19.56	0.59	32	0.69	37.33	0.72	42.67	0.78
SA1	2	427	0.06	**4.83**	0.07	2.43	0.21	1.30	0.40	0.80	0.89	0.74	1.53	0.93	2	1.08	2	1.04

Data are presented as average of three analyses, each performed in triplicate.

*S. aureus Staphylococcus aureus*, *MIC* minimum inhibitory concentration, *FICI* fractional inhibitory concentration index, *OXA* oxacillin.

Bold values: antagonism (ƩFIC > 4).

A strong antagonistic effect, manifested by very high FIC indices of 8.56 (clinical isolate SA1) and 8.51 (MSSA ATCC 29213), was observed for *A. vulgare* oil at a concentration 2048 µg/mL, when combined with oxacillin. At the same concentration, *A. vulgare* oil caused an adverse interaction in MRSA ATCC 43300 with a FICI of 5.78. This antagonistic effect was also observed at a concentration of 1024 µg/mL with the MSSA clinical isolate, SA1 (FICI 4.30), and the reference strain, ATCC 29213 (FICI 4.27). Indifferent relationships were displayed in all other combinations for both agents. Interestingly, a significant increase in MIC of oxacillin was observed when combined with *A. vulgare* oil at two lowest concentrations tested (32 and 16 µg/mL). In these cases, the MIC values rose by approximately 1/3 and 3/4, respectively, when compared to the MIC of the antibiotic alone, against MSSA ATTC 29213. In combination with oxacillin, *C. nucifera* and *E. guineensis* oils also exerted antagonistic properties in all tested strains, at a concentration of 2048 µg/mL (FICI 4.02–6.28). *C. nucifera* oil showed the strongest antagonistic effect for MRSA ATCC 43300 strain, followed by MRSA clinical isolate SA1 and MSSA ATCC 29213 strain (FICI values were 6.28, 6.03, and 4.09, respectively). *E. guineensis* oil exerted similar antagonistic activity in MSSA ATCC 29213, MRSA clinical isolate SA1, and MRSA ATCC 43300 strain, with FICI values of 4.88, 4.83, and 4.02, respectively. The rise of oxacillin MIC, when combined with the lowest concentrations (16–64 µg/mL) of the tested oils, was even more pronounced with *C. nucifera*. It induced an increase of MIC in MSSA ATCC 29213 strain up to more than two times. A slight increase of oxacillin MIC was also detected in ATCC 29213 strain at a concentration of 0.32 µg/mL of *E. guineensis* oil.

### Antagonistic growth-inhibitory effect of lauric acid with oxacillin against *S. aureus*

As mentioned above, GC-FID analysis revealed LA to be the predominant fatty acid in all tested oils. Based on this result, the susceptibility of nine *S. aureus* strains to oxacillin, LA, and a combination of oxacillin and LA was investigated using the same methodology as that used for palm oils. As shown in Table 3, MIC values of oxacillin ranged from 0.53 to 683 µg/mL. MIC values of LA were the same for all *S. aureus* strains at 256 µg/mL, except for MRSA ATCC 43300, where the MIC of LA decreased to 242 µg/mL.

**Table 3:** Combinatory effect of lauric acid and oxacillin against *Staphylococcus aureus* determined by checkerboard method.

*S. aureus* strain	MIC of compounds alone (µg/mL)		OXA with LA at concentration (µg/mL)															
			1024		512		256		128		64		32		16		8	
	MIC OXA	MIC LA	MIC OXA	FICI	MIC OXA	FICI	MIC OXA	FICI	MIC OXA	FICI	MIC OXA	FICI	MIC OXA	FICI	MIC OXA	FICI	MIC OXA	FICI
29213	0.53	256	0.01	**4.01**	0.01	2.01	0.01	1.02	0.58	1.61	0.67	1.51	0.78	1.6	0.89	1.75	0.78	1.5
33591	683	256	8	**4.01**	8	2.01	8	1.01	319.56	0.97	853	1.5	1024	1.63	1024	1.5	1024	1.53
33592	569	256	8	**4.01**	8	2.01	8	1.01	112.89	0.7	512	1.15	682.49	1.33	568.89	1.06	512	0.97
43300	46.22	242	2	**4.28**	2.22	2.17	2.67	1.12	9.11	0.73	33.78	1	103.11	2.36	110.22	2.45	67.56	1.49
EMRSA 15	99.56	256	1	**4.01**	1	2.01	1	1.01	8.22	0.58	112	1.38	> 128	> 1.41	128	1.35	128	1.32
BAA 976	32	256	0.83	**4.03**	0.83	2.03	0.83	1.03	19.56	1.11	85.33	2.92	128	**4.13**	118.86	3.78	64	2.03
SA1	1.44	256	0.13	**4.09**	0.13	2.09	0.13	1.09	0.39	0.77	1	0.94	1.78	1.36	1.89	1.37	2.22	1.57
SA2	67.56	256	2	**4.03**	2	2.03	7.11	1.11	24	0.86	74.64	1.36	92.44	1.49	85.33	1.33	74.67	1.14
SA3	0.47	256	0.06	**4.13**	0.06	2.13	0.06	1.13	0.5	1.56	0.83	2.01	0.61	1.24	1.17	2.53	0.69	1.49

Data are presented as average of three analyses, each performed in triplicate.

*S. aureus Staphylococcus aureus*, *MIC* minimum inhibitory concentration, *FICI* fractional inhibitory concentration index, *OXA* oxacillin, *LA* lauric acid.

Bold values: antagonism (ƩFIC > 4).

Subsequently, FICI values were calculated and the effect of combinations was analysed. Antagonistic mode of action was observed for LA at a concentration of 1024 µg/mL, when combined with oxacillin (FICI values were 4.01–4.28) in all tested *S. aureus* strains; the strongest antagonistic interaction was exhibited for the MRSA ATCC 43300 strain. Other combinations of concentrations revealed indifferent relationships, except for a combination of LA at 32 µg/mL with oxacillin against the *S. aureus* ATCC BAA 976 strain. In this case, the MIC of oxacillin increased four times, to 128 µg/mL, and the FICI value was 4.13. In all tested bacterial strains, rise of MIC values of tested antibiotics occurred when combined with LA at certain concentrations (16–256 µg/mL), up to two times with the average increase by nearly two-thirds.

**Figure 1:**
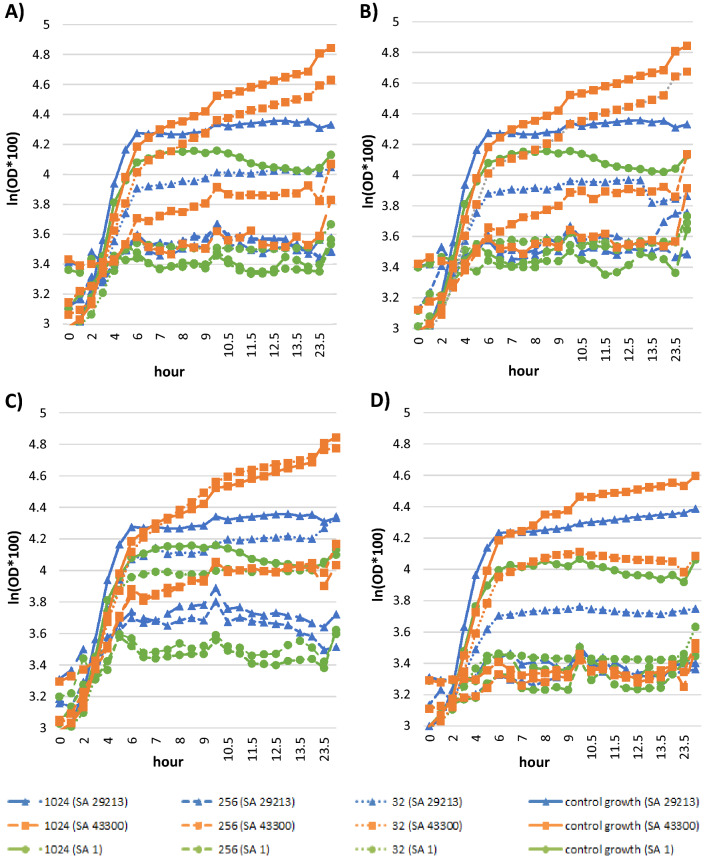
Growth curves of *Staphylococcus aureus* strains upon different concentrations of hydrolyzed palm oils or lauric acid ((**A**)—*Astrocaryum vulgare* oil; (**B**)—*Cocos nucifera* oil; (**C**)—*Elaeis guineensis* oil; (**D**)—lauric acid) determined spectrophotometrically. *SA Staphylococcus aureus*, *OD* optical density.

To determine whether the hydrolyzed seed oils of *A. vulgare*, *C. nucifera*, and *E. guineensis* and LA influence the growth of *S. aureus* strains, the analysis of growth curves of three *S. aureus* strains, namely ATCC 29213, ATCC 43300, and clinical isolate SA1, was performed. According to Fig. 1, the graphical evaluation of bacterial growth upon increasing concentrations of hydrolyzed oils and LA revealed decreasing growth rate and increase in generation time for all tested *S. aureus* strains.

## Discussion

In our study, crude seed oils rich in MCFAs, hydrolyzed by porcine pancreatic lipase, and LA showed in vitro growth-inhibitory effect against reference strains and clinical isolates of *S. aureus*. As per the results of GC analysis, the oils used for antibacterial testing were of standard composition, as reported by other groups, who also identified LA as the major constituent of *A. vulgare*^48^, *C. nucifera*^49^, and *E. guineensis* oils^50^. In this study, the MIC values of LA against the tested *S. aureus* strains (242–256 µg/mL) were comparable to those reported by other authors. For example, Batovska et al.^42^ reported an MIC values for LA, measured by the macrodilution method against *S. aureus* strains at ≥ 125 µg/mL. Moreover, Nitbani et al.^51^ reported the antibacterial activity of LA isolated from *C. nucifera* oil against *S. aureus*. In contrast, Parsons et al.^52^ determined an MIC value of 50 µg/mL (250 µM) for LA against *S. aureus*, using the broth microdilution method. Similarly, Kelsey et al.^53^ observed MIC values equal to 50 µg/mL of LA against three different *S. aureus* strains, using turbidimetry with visual evaluation and ethanol as a solvent; however, these variations could be caused by the different methodologies and *S. aureus* strains used. To the best of our knowledge, there is only limited information on the antibacterial properties of palm oils rich in MCFAs. Free-fatty acids are known to have antibacterial activity in contrast with those bound to triglycerides^27^. As described by various authors previously^46,52^, the antistaphylococcal effect of fatty acids is induced by disruption of bacterial cell membrane resulting in its destabilization by inducing tubule formation on the lipid bilayer and cell lysis; thus, the antimicrobial activity of oils containing fatty acids is facilitated only upon hydrolysis of triglycerides^26–28^. In contrast, Ubgogu^43^ showed that *E. guineensis* oil exerted slight antibacterial effect on *S. aureus,* without getting hydrolyzed, using the disc diffusion technique. This result is the opposite of that observed in this study, where the oils acted as an antibacterial only after being hydrolyzed by the porcine pancreatic lipase. Rossato et al.^54^ also observed no antistaphylococcal activity of unhydrolyzed *A. vulgare* oil. Results of in vitro screening of *C. nucifera* oil and other MCFA-containing fats, with lipolytic enzymes that simulated gastric conditions in piglets, showed a significant change in suppression of gut microbiota (total anaerobes and *E. coli*)^55^. This finding suggests that MCFA-rich oils exert their antibacterial effects after enzymatic hydrolysis by lipases synthesized in the gastrointestinal tracts of humans and other animals.

Studies on the combinatory effect of MCFAs and their esters with different organic acids, inorganic compounds, and antibiotics against various bacteria, including *S. aureus*, can be found in literature^26,42,47,56^. However, the findings of these studies markedly vary, depending on the class of antimicrobial agent used. For example, Kitahara et al.^47^ observed synergistic interaction of LA at 50 µg/mL with gentamicin (FICI values were 0.25–0.31) and imipenem (0.13–0.25) and indifferent interactions of LA (50 µg/mL) with ampicillin and oxacillin, against MRSA clinical isolates. Similarly, Hess et al.^57^ observed indifferent interactions of the LA-ampicillin or vancomycin combination against *S. aureus* biofilms and a synergistic interaction between LA and streptomycin. We observed indifferent antistaphylococcal action of oxacillin and LA (50 µg/mL); however, increasing the amount (≥ 1024 µg/mL) of LA, produced strong antagonism in the presence of the antibiotic. The reason for the lack of antagonism in Kitahara et al.^47^ and Hess et al.^57^ could be the concentration of LA used in the study, which probably did not reach a sufficient value for the antagonism to be exerted. There is no information on the interaction of palm oils rich in MCFAs as their antibacterial activity was not confirmed in the study^54,58^ because hydrolysis of oils was not performed^26^.

From the results presented in this study, it is not possible to infer by which mechanism the antagonism between LA, tested oils and oxacillin occurs. Nevertheless, the detected prolongation of the generation time resulting in the decrease of specific growth rate arising with increasing concentrations of LA and LA-rich tested palm oils indicates that a possible mechanism underlying the antagonistic interactions between tested compounds might lie in the halting of cell division caused by LA/*A. vulgare*/*C. nucifera*/*E. guineensis* oil. It can be assumed that while the LA temporarily prevents the growth of bacterial cells, oxacillin is not able to properly exert its activity. However, it is not just the membrane lipid structure that can change under the influence of exogenous lipids but the protein structure as well. Other factors including membrane strain may account for the organization of membrane proteins^59^. It can be hypothesized that under increased membrane strain, such as after the treatment of lipid bilayers with high concentrations of LA^46^, membrane protein function is altered^60^. Such a circumstance can affect the antibacterial activity of oxacillin, which strongly depends on its ability to inhibit bacterial cell wall synthesis by preferentially binding to penicillin-binding proteins (PBPs) that are located inside the bacterial cell wall^61^. Therefore, oxacillin probably becomes ineffective after change in PBP function, induced by LA. As membrane protein stability also depends on membrane energetics, LA can reduce membrane fluidity and disrupt the electron transport system, perhaps by restricting the movement of carriers within the membrane^44^. The eventual impairment of membrane electron carriers can lead to a change in the intracellular and extracellular pH, which can cause the precipitation of PBPs^62^ and make them to lose the ability to interact with oxacillin. In addition, the change in extracellular pH can affect chemical structure of oxacillin as this drug is highly unstable in acidic environments^63^. Moreover, *S. aureus* is known to produce persisters, which are representing a fraction of the bacterial population that exhibits tolerance to antibiotics in response to various stresses^64^. According to the finding of Peyrusson et al.^65^, *S. aureus* in the presence of high concentration of various antibiotics including oxacillin showed a biphasic killing manner, meaning that a bulk of the bacterial population was susceptible and rapidly killed while a subpopulation with a slower killing rate was persisting for a much longer period of time, in addition showing the reversibility of the phenotype after antibiotic removal. Therefore, another possible explanation of antagonism between oxacillin and LA/LA-rich palm oils, can be in inducing persister cells of *S. aureus* in the presence of high concentrations of tested compounds.

Food-drug interactions are a major threat to safe and effective oral pharmacotherapy and can result in decreased bioavailability of a drug, which predisposes the patient to treatment failure, increases the risk of adverse events, and may even precipitate toxicities^19,66^. For this reason, coadministration of a drug with specific foods is noted in medical leaflets. Generally, food intake can influence the effectiveness of an antibiotic^67^. Ingestion of food, dietary fiber, or milk reduces the bioavailability of most antibiotics, including some penicillins^68^. For example, minerals in milk and cheese create complexes with antibiotics that decrease their absorption^69^, and as seen in the case of isoxazolyl penicillins, when administered shortly before or after a meal, delayed gastric emptying and increased acidity interfere with their absorption^70^. The consumption of coconut oil and related products is currently growing among certain populations, for the claimed health benefits associated with cardiovascular disease and weight loss^71^. On the other hand, recommendations of lowering intake of saturated fatty acids and replacing them with unsaturated fatty acids exist in order to reduce risk of atherosclerosis and type-2 diabetes^72,73^. The average concentration of LA in human serum of healthy adult male and female blood donors, ages ranging from 18 to 55 years, was found to be < 10 µg/mL^74^. It has, however, been proven that higher (14.2–140 g/day) intakes of MCFAs in diet may result in higher concentrations of high density lipoprotein cholesterol than found with long-chain fatty acids, highlighting the importance of considering chain length when measuring the effect of dietary saturated fatty acids on lipid profile^75^. Moreover, in a high-carbohydrate, high-fat diet, the increases in systolic blood pressure and diastolic stiffness in the heart were inhibited in mice with diet enriched by virgin coconut oil, composed predominantly of LA, at concentrations of 200 g/kg^76^. Our results showing strong in vitro antagonistic effect of oxacillin with LA or LA-rich palm oils at concentrations ≥ 1024 µg/mL (meaning approximately ≥ 1.113 g/kg; 1 g/kg of body mass) suggest that simultaneous administration of these agents can negatively affect their pharmacological properties. The recommended intake of fats in the diet of men is estimated to be around 65 g/day (daily energy intake 2000 kcal with fats representing 30% of it)^77^. According to FAO^78^, average energy requirement of an adult female is 2410 kcal/day and of an adult men is 3100 kcal/day. Counting daily intake of fats as 30% of total energy requirement, the daily intake of fats can be estimated to be 80 g/day for women and 103 g/day for men. Studies on coconut oil supplementation in diet usually focus on the addition of the oil in range 20–50 g/day^79–81^. Nevertheless, there have been reports on even higher daily consumption of coconut oil reaching up to 80 g/day^82–84^. Therefore, the observed antagonistic action between LA-rich oils/LA may be important especially in high-fat diets. Thus, our results showing strong in vitro antagonistic effect of oxacillin with LA or LA-rich palm oils suggest that simultaneous administration of these agents in high, but still reachable concentrations can negatively affect their pharmacological properties in the treatment of *S. aureus*. The risk of antagonistic interactions between oxacillin and LA-rich oils might be primarily important to systemic application, as their antibacterial effect is attributed to fatty acids unleashed from triglycerides only, therefore the topical application of LA-rich oils such as *A. vulgare*/*C. nucifera*/*E. guineensis* should not influence the antibacterial activity of oxacillin. But, according to Verallo-Rowell et al.^85^, 5 mL of extra virgin coconut oil applied two times a day on the affected areas that include the test sites is able to decolonise skin from *S. aureus* in adults with atopic dermatitis. This discrepancies between the theoretical background and practice can be debited to the lipolytic activity of skin microbiota, including staphylococci^86^, highlighting the importance of possible negative effect of LA-rich oils on topical treatment of *S. aureus*. Moreover, LA was previously tested in vitro at concentration of 0.24–500 μg/mL to evaluate its antibacterial properties against various bacteria causing inflammatory acne vulgaris, including *S. aureus*, proposing it as an promising remedy in the treatment of staphylococcal skin infections^87^. However, the mentioned tested concentrations of LA were not high enough for the antagonism with oxacillin to be exerted. Nevertheless, these hypotheses must be confirmed by further in vitro and in vivo tests and clinical trials because physiological processes can also induce changes in antibacterial activity of tested compounds.

In summary, this in vitro study revealed a concentration-dependent antagonistic effect between *A. vulgare*, *C. nucifera*, and *E. guineensis* oils when combined with oxacillin in higher amounts against various strains of *S. aureus*. The strongest antagonism was observed for *A. vulgare* oil, which contains the highest amount of LA. This compound was identified as the main agent responsible for antagonistic antistaphylococcal action of all oils assayed. To the best of our knowledge, this is the first study to report the antagonistic interactions between these agents. The mechanism underlying the antagonistic action of tested agents probably acts at the cellular level and is linked to the cell membranes. These findings suggest that interference between oxacillin and palm oils and their constituents can negatively affect the treatment of staphylococcal infections in humans and animals. However, these assumptions are based on in vitro tests and the negative interactions of the above-mentioned combinations should be confirmed by in vivo trials.

## Methods

### Chemicals and samples preparation

LA, *C. nucifera* and *E. guineensis* oils, and oxacillin sodium monohydrate were obtained from Sigma-Aldrich (Prague, CZ). *A. vulgare* oil was purchased from Sweet Natural Botanicals (Panama City, FL, USA). LA and *A. vulgare*, *C. nucifera*, and *E. guineensis* oils were dispersed in dimethyl sulfoxide, which was previously proposed for sparingly soluble substances^88^, including the LA^89^ and palm oil^90^; and emulsified using Tween 80 (Sigma-Aldrich, Prague, CZ), that is a common procedure of hydrophobic sample preparation^91^, not influencing the antibacterial properties of tested compounds when used in recommended concentrations^92,93^. Complete dissolution of LA was achieved by heating (70 °C for 10 min) it in an ultrasonic-bath. Analysed plant oils were selected based on the high content of LA as described in literature^94–96^. Hydrolysis of oils, to induce antimicrobial properties, was achieved by adding 5 × 10^–3^ mg/mL of porcine pancreatic lipase (Sigma-Aldrich, Prague, CZ) and shaking for 1 h in a water-bath heated to 37 °C. Degree of hydrolysis of oils was dependent on the lipolytic activity of the enzyme. One unit of porcine pancreatic lipase hydrolyzes 1.0 microequivalent of fatty acid from triacetin in 1 h at pH 7.4, at 37 °C.

### Bacterial strains and media

In this study, nine *S. aureus* strains were tested. Five reference strains including methicillin sensitive (MSSA) ATCC 29213, MRSA ATCC 33591, ATCC 33592, ATCC 43300 and ATCC BAA 976 were purchased from Oxoid (Basingstoke, UK). Three clinical isolates of drug resistant *S. aureus* (SA1, SA2, and SA3) and one epidemic MRSA strain (EMRSA-15) were obtained from the Motol University Hospital (Prague, CZ). Oxacillin, gentamicin, tetracycline, and penicillin were used as markers of the antibiotic resistance as it has been previously determined^97–99^. Based on the MIC values, clinical isolates were identified to be resistant to: SA1—resistant to gentamicin (MIC 8 µg/mL) and tetracycline (MIC 8 µg/mL); SA2—resistant to oxacillin (MIC 68 µg/mL), gentamicin (MIC 16 µg/mL) and tetracycline (MIC 8 µg/mL); SA3—resistant to gentamicin (MIC 8 µg/mL) and penicillin (MIC 18.67 µg/mL); EMRSA-15—resistant to oxacillin (MIC 99.56 µg/mL) and penicillin (MIC 16 µg/mL). The clinical isolates were identified using matrix-assisted laser desorption/ionization time-of-flight mass spectrometry (MALDI-TOF MS), as described previously by Rondevaldova et al.^98^. Bacterial stocks were stored at 4 °C on Müller–Hinton agar (Oxoid, Basingstoke, UK). Working cultures were maintained in Müller-Hinton broth at 37 °C for 24 h before testing.

### Determination of fatty acid composition

To evaluate fatty acid composition of oils obtained from *A. vulgare*, *C. nucifera*, and *E. guineensis*, alkaline trans-methylation of fatty acids was carried out as described by Raes et al.^100^. Analysis of methyl esters was performed using gas chromatography (GC) with the HP 6890 chromatograph (Agilent Technologies, Inc., Santa Clara, CA, USA), equipped with a 60 m DB-23 capillary column (60 m × 0.25 mm × 0.25 μm) and a flame-ionization detector (FID); split injections were performed using an Agilent autosampler. A total of 1 µL of standards in hexane were injected in split mode (1:40 ratio) into the injector, heated to 230 °C. The column temperature was initially set at 120 °C for 6 min then programmed to 170 °C at a rate of 15 °C/min. The temperate gradient was further configured to 210 °C at the rate of 3 °C/min and held for 13.5 min. Finally, the temperature was programmed to 230 °C at the rate of 40 °C/min and held for 7 min. Nitrogen was used as the carrier gas, at a flow rate of 0.8 mL/min. Supelco 37 Component FAME Mix, PUFA 1, PUFA 2, PUFA 3, *trans*-vaccenic acid, and a mixture of conjugated isomers of linoleic acid (Sigma-Aldrich, Prague, CZ) were used as standards. Fatty acids were identified based on retention times with respect to standards.

### Evaluation of minimum inhibitory concentrations and antagonistic combinatory effect

Using guidelines of the Clinical and Laboratory Standards Institute^101^, the antibacterial activities of oxacillin; oils extracted from *A. vulgare*, *C. nucifera*, and *E. guineensis*; and LA were evaluated in vitro by the broth microdilution method, modified as per the recommendations of Cos et al.^102^, for effectively assessing the anti-infective potential of the natural products. Antistaphylococcal effect of a combination of oxacillin/LA or oxacillin/palm oil was tested in vitro using the microdilution broth checkerboard method, as described in the Clinical Microbiology Procedures Handbook^103^. The determination of MIC of oxacillin, palm oils and LA, as well as oxacillin/LA or oxacillin/*A. vulgare* oil*/C. nucifera* oil/*E. guineensis* oil combinatory effect evaluation by FICI was performed in 96-well microtiter plates. For the testing of combinatory effects, eight twofold serial dilutions of oxacillin were placed in the horizontal rows of the plate and were subsequently cross-diluted vertically by eight twofold serial dilutions of the test compound (palm oil or LA), resulting in 64 different combinations of concentrations. The initial concentration for palm oils was 4096 µg/mL and for LA 2048 µg/mL; the starting concentration of antibiotic was adjusted according to the tested strain. The microtiter plate assay was performed using the automatic pipetting platform, Freedom EVO 100 equipped with a four-channel liquid handling arm (Tecan, Männedorf, CH). Cation-adjusted Müller-Hinton broth, equilibrated to a final pH of 7.6 with Trizma base (Sigma-Aldrich, Prague, CZ) was used as growth medium. Buffering the culture media was performed to ensure the stability of oxacillin that is known to decrease under the low pH conditions^63^. Inoculation of the plates was carried out using bacterial suspensions, at a final density of 5 × 10^5^ CFU/mL, standardized using Densi-La-Meter II by adjusting turbidity of the microorganism suspension to 0.5 McFarland standard. Next, incubation at 37 °C for 24 h was performed. Evaluation of bacterial growth was performed spectrophotometrically using multimode reader Cytation 3 (BioTek Instruments, Winooski, VT) at 405 nm. MIC values were expressed as the lowest compound concentrations that resulted in ≥ 80% growth reduction compared to that of the agent-free growth control. The lipase added to the Müller-Hinton broth, in concentrations ranging from 0.005 to 9.77 × 10^–6^ mg/mL did not affect the growth of any strain of *S. aureus* tested when assayed as a negative control. FICI values were determined as ΣFIC, derived from the equation,

$$\Sigma\text{ FIC}= {\text{FIC}}_{\text{A}}+{\text{FIC}}_{\text{B}}$$

where, FIC_A_ $=\frac{{\text{MIC}}_{\text{A }}(\text{in \,the\, presence \,of\, B})}{{\text{MIC}}_{\text{A}}(\text{alone})}$ and FIC_B_ $=\frac{{\text{MIC}}_{\text{B}} (\text{in \,the\, presence\, of \,A})}{{\text{MIC}}_{\text{B}}(\text{alone})}$.

According to the value of FIC, three different effects could be observed according to Odds^104^—synergy (ΣFIC ≤ 0.5), indifference (ΣFIC > 0.5–4), and antagonism (ΣFIC > 4). All compounds and their combinations were tested in three independent experiments, each carried out in triplicate; MIC values and FICs presented in this paper are average values.

### Growth rates determination

To evaluate the influence of tested palm oils and LA concentrations on parameters of the *S. aureus* growth, standardised microdilution assay was used^105^. Briefly, the determination in 96-well microtiter plates where eight two-fold serial dilutions of tested compound (LA or *A. vulgare*/*C. nucifera/E. guineensis* oil emulsion cleaved by porcine pancreatic lipase as described previously) starting at concentration 4096 µg/mL in cation-adjusted Müller-Hinton broth was performed. Next, the plates were inoculated with bacterial suspensions at a final density of 5 × 10^5^ CFU/mL, standardized using Densi-La-Meter II by adjusting turbidity of the microorganism suspension to 0.5 McFarland standard, same as in case of MIC and FIC determination. Subsequently, incubation at 37 °C was performed, measuring the absorbance of each well spectrophotometrically by multimode reader Cytation 3 (BioTek Instruments, Winooski, VT) at 405 nm every half-to-one hour for 14 h and after 24 h.

## Data Availability

The datasets generated during and/or analysed during the current study are available from the corresponding author on reasonable request.
